# Aging and Old Age in Colombian Trans Women: A Grounded Theory Approach

**DOI:** 10.1093/geroni/igaa057.991

**Published:** 2020-12-16

**Authors:** Maria Reyes, Maldonado Daniela, Méndez Carlos, Maria Ariza, Vannesa Arias, Isabella Pachon

**Affiliations:** 1 Universidad El Bosque, Bogota, Colombia; 2 Red Comunitaria Trans, Bogota, Distrito Capital de Bogota, Colombia; 3 Universidad El Bosque, Bogota, Distrito Capital de Bogota, Colombia; 4 Universidad El Bosque, BOGOTA, Distrito Capital de Bogota, Colombia

## Abstract

Trans people around the world represent one of the most marginalized and stigmatized groups in society who are at high risk of discrimination, violence and abuse (White Hughto, Reisner, & Pachankis, 2015). In Colombia, older adults face a situation of vulnerability and poverty, and this situation is even more dramatic for older people with diverse gender identities. The research focused on understanding the challenges that a group of Colombian trans women experience in the process of aging and old age. An exploratory qualitative research project was carried out using constructionist Grounded Theory. Twenty five trans-women from 50 to 67 years old who live in Bogotá, Colombia participated. The data were collected using semi-structured interviews. The results address three main research questions: (a) How the participants overcome the life expectancy and achieve middle and older adulthood? (b) What are the barriers the participants faced in the aging process? (c) How do the group of Colombian trans women imagine and considered a successful aging? The results evidenced that the process of aging of the participants was influenced by six psycho-socio-cultural categories. A central category that was identified as opportunity, which was influenced by five categories: a) Violence, discrimination and transphobia, b) Transit process, c) Personal strengths d) Mobilization and activism and e) experience and perception the old age. Discussion. The challenges that the participants experienced were those associated with the process of aging and to their gender identity. Trans women achieve middle and old adulthood for their personal strengths, activism and mobilization.

